# A Rare Diagnosis of Primary Effusion Lymphoma Presenting With Three-Cavity Effusion: A Case Report

**DOI:** 10.7759/cureus.89430

**Published:** 2025-08-05

**Authors:** Hari Movva, Yasamin Rastgar, Kinan Obeidat, Alexandra Lewis

**Affiliations:** 1 Internal Medicine, University of Texas Medical Branch at Galveston, Galveston, USA; 2 Medical School, University of Texas Medical Branch at Galveston, Galveston, USA; 3 Internal Medicine, Baylor College of Medicine, Houston, USA

**Keywords:** anti-retroviral therapy, effusion, hiv aids, immunocompromised patient, primary effusion lymphoma, three-cavity effusion

## Abstract

We describe a 50-year-old incarcerated transgender female with advanced human immunodeficiency virus (HIV)/acquired immunodeficiency syndrome (AIDS) who was not compliant with antiretroviral therapy (ART). She presented with a three-cavity effusion (peritoneal, pleural, and pericardial) complicated by superimposed spontaneous bacterial peritonitis (SBP). Cytologic smears, flow cytometry, and immunostaining revealed primary effusion lymphoma (PEL). This case report aims to provide a clinical presentation of PEL and offers an overview of its diagnosis and current management strategies.

## Introduction

Primary effusion lymphoma (PEL) was first described in the literature in 1989 in a 31-year-old male patient with HIV who presented with a malignant pleural effusion interpreted as a probable large cell lymphoma, but of which the exact cause was unknown at the time [1]. Since then, much has been elucidated regarding PEL's unique biological and clinicopathological characteristics. PEL is a rare and aggressive subtype of mature B-cell non-Hodgkin lymphoma (NHL), typically described in patients with HIV, but is also associated with other causes of immune suppression [2]. It classically presents as lymphomatous effusions of serous body cavities without associated mass lesions in immunocompromised patients infected with Kaposi sarcoma herpesvirus/human herpesvirus-8 (KSHV/HHV8), a virus commonly seen in PEL, with or without Epstein-Barr virus (EBV) coinfection, a virus commonly seen in immunosuppression [2-4]. The effusions seen in PEL commonly present within one to two body cavities, and few cases have been described with effusions in three body cavities [2-7]. Given its rarity, there is importance in gaining a greater understanding of PEL within clinical medicine. We herein report a case of classical PEL involving three body cavities with superimposed spontaneous bacterial peritonitis (SBP) in an incarcerated patient with advanced HIV.

## Case presentation

A 50-year-old incarcerated transgender patient (male to female) with a past medical history significant for HIV off antiretroviral therapy (ART) for one year, hypertension (HTN), cerebrovascular accident (CVA), hyperlipidemia (HLD), and asthma presented as a transfer from an outside emergency department for diffuse abdominal pain, intractable nausea, and vomiting for one day and a 55-pound weight loss over the preceding two months. She had been HIV positive 20 years prior to presentation and had not been adherent to ART for one year after a reported disagreement with her previous provider. Notably, patient records showed severe immunosuppression two years prior to presentation (CD4% of 8% and absolute CD4 of 79 cells/mm^3). The patient had a non-contributory surgical and family history and denied tobacco, alcohol, or intravenous (IV) drug use. She took daily *Mycobacterium avium* complex (MAC) and *Pneumocystis* pneumonia (PCP) prophylaxis prior to admission.

Physical exam showed a blood pressure (BP) of 103/72 mmHg, heart rate (HR) of 142 beats/minute, body mass index (BMI) of 16 kg/m2, and the patient was afebrile. EKG was significant for sinus tachycardia. She was ill-appearing and cachectic, with an abdominal exam showing prominent distension, positive fluid wave, and diffuse tenderness. Ascites was appreciated on bedside ultrasound, and pertinent lab findings are described (Table 1).

**Table 1:** Patient laboratory values and reference ranges NT-pro-BNP: N-terminal pro-B-type natriuretic peptide

	Patient Value	Reference Range
Hemoglobin (Hgb)	7.5 g/dL	12.1 – 15.1 g/dL
Mean Corpuscular Volume (MCV)	101.3 fL	80 – 100 fL
Platelets	108,000 /µL	150,000 – 450,000 /µL
NT-pro-BNP	1,940 pg/mL	< 450 pg/mL
Thyroid Stimulating Hormone (TSH)	6.11 uIU/mL	0.4 – 4.0 mIU/L
HIV1 Viral Load	3.4 million copies/ml	-
CD4 Percentage	2%	30 – 60%
CD4 Absolute Count	8 cells/mm^3^	500 – 1,500 cells/ mm^3^
Ceruloplasmin	46 mg/dL	20 – 60 mg/dL
Anti-smooth muscle antibody	14 IU/mL	-
Anti-mitochondrial antibody	5.6 IU/mL	-
Alpha-fetoprotein (AFP)	0.9 ng/mL	< 10 ng/mL

**Figure 1:**
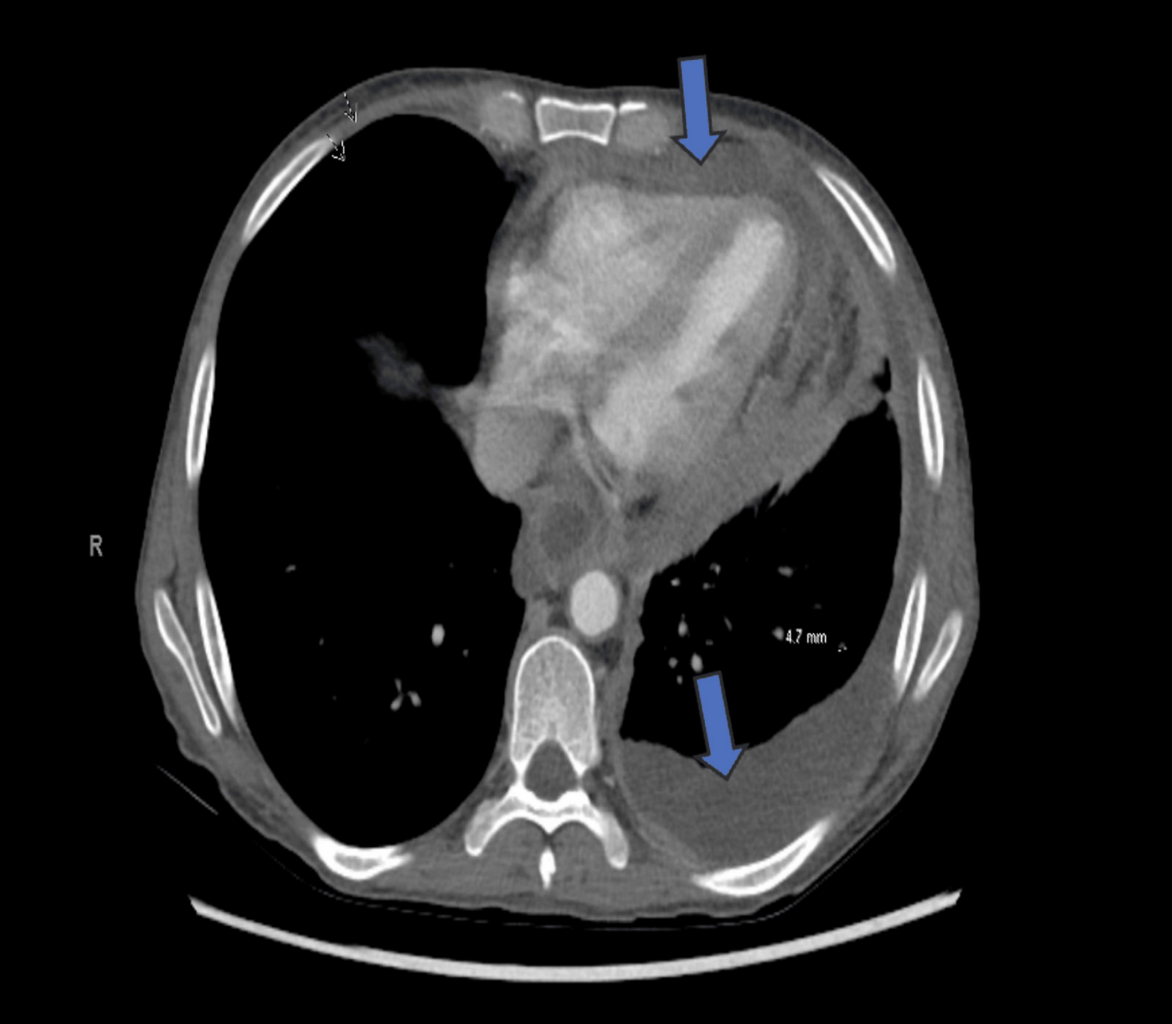
CT Thorax showing pericardial effusion and left-sided pleural effusion.

**Figure 2:**
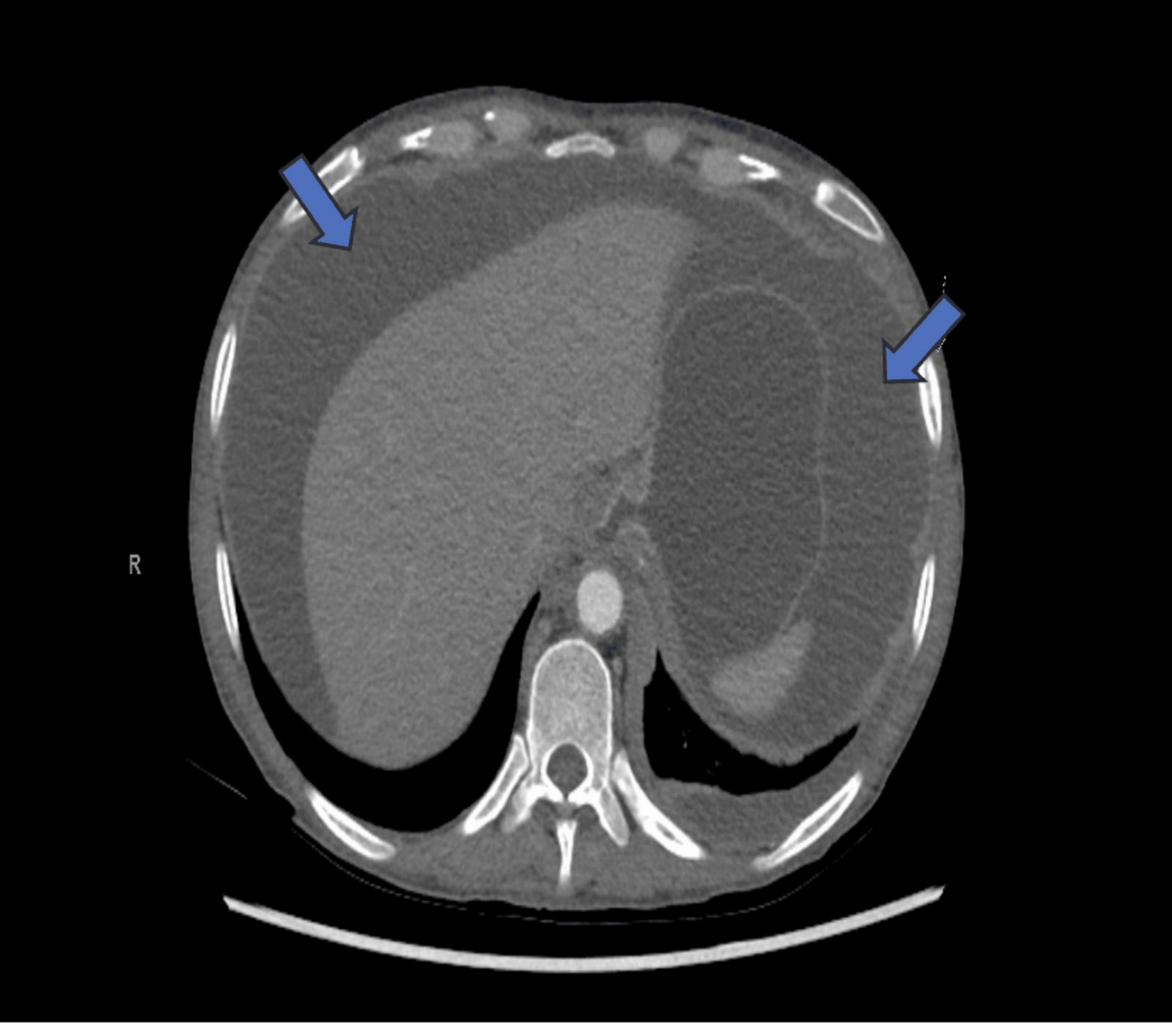
CT Abdomen showing large-volume abdominal ascites with normal appearing liver.

Hepatitis serologies, antinuclear antibody (ANA), Quantiferon-TB Gold, Fungitell, serum cryptococcal antigen, and *Histoplasma* in serum and urine were negative, and syphilis was non-reactive. Computed tomography (CT) of her abdomen, pelvis, and thorax showed large-volume abdominal and pelvic ascites, a moderate left-sided pleural effusion, and a pericardial effusion without evidence of liver cirrhosis or any detectable tumor masses (Figures 1, 2). Pleural fluid analysis met Light’s Criteria for an exudative effusion (lactate dehydrogenase (LDH) > two-thirds the upper limit of normal), and ascitic fluid analysis raised concern for SBP, given an elevated white blood cell count and cultures showing *E. coli* and *K. pneumoniae* coinfection (Table 2). The patient was subsequently started on antibiotics.

**Table 2:** Microbiological analysis of pleural and ascitic fluids.

	Pleural Fluid	Ascitic Fluid
Appearance	Light yellow	Yellow
Lactate Dehydrogenase (U/L) (Body Fluid)	> 5,000	-
Lactate Dehydrogenase (U/L) (Serum)	323	-
Protein (g/dl) (Body Fluid)	4.5	4.4
Protein (g/dl) (Serum)	5.9	5.9
White Blood Count (/uL)	7,469	2,296
Red Blood Count (/uL)	< 3,000	< 3,000
Body Fluid Polymorphonuclear Leukocytes (%)	-	-
Body Fluid Other Cells (%)	99%	89%
Body Fluid Number of Cells Counted (cells/uL)	100	100

Cytologic smears of the ascitic and pleural fluid showed large neoplastic cells with immunoblastic/plasmablastic morphology and multiple atypical mitoses. Flow cytometric analysis was positive for the expression of CD38, CD45 (dim), CD81, and CD138, with negative expression of T-cell markers, pan-T antigens, and kappa and lambda light chains. Immunostaining of the atypical lymphoid cells showed co-expression of HHV-8, MUM1, and epithelial membrane antigen (EMA). These markers are commonly positive in PEL and help distinguish it from other forms of lymphoma. *In situ* hybridization showed EBV coinfection. A diagnosis of PEL was made with these findings one day after admission. Given the poor prognosis, a goals of care discussion was initiated with the patient, and she elected for hospice care and passed away four days after diagnosis.

## Discussion

While the occurrence of PEL in patients with HIV is not rare, the involvement of three body cavities makes this case unique. The patient’s immunocompromised state, coupled with low CD4 counts, made her more susceptible to malignancy. The presence of ascites was found to be unrelated to cirrhosis, as exemplified by imaging and laboratory tests revealing no involvement of the liver, and instead was due to neoplasm and infection.

Among patients with HIV, it has been estimated that 25-40% will go on to develop malignancy. Of these, 5-20% will be because of NHL, and fewer than 5% will be PEL [8-11]. Despite the relative commonality in HIV-positive patients with PEL, the prognosis remains poor. Most cases end with patient death within the year, even when considering the use of effective combination ART. Factors that improve prognosis include extracavitary involvement and EBV positivity [12].

The classical symptoms of peritoneal effusion include abdominal distention, abdominal pain, nausea, vomiting, and dyspnea, similar to the presentation of this patient. Physical exam could reveal shifting dullness and a positive fluid wave test, consistent with this case. The symptoms of a pericardial effusion involve shortness of breath, orthopnea, retrosternal chest pain, and compressive symptoms like hoarseness, nausea, dysphagia, and hiccups. The symptoms of a pleural effusion involve dyspnea, shortness of breath, cyanosis, tachypnea, confusion, pleuritic chest pain, and nonproductive cough [13,14]. The patient was not exhibiting signs of effusions in the pericardial or pleural cavities, but these were found to have significant fluid accumulation on imaging. Cytologic studies confirmed PEL was the underlying cause. The patient was found to have effusions in three classic effusion sites, whereas most patients involve a single cavity space.

Due to its rarity, the treatment regimens of PEL vary with each patient. Medication regimens involving cyclophosphamide, doxorubicin, vincristine, and prednisolone, but the prognosis remains poor. Antiviral medications have been used either alone or in combination with chemotherapy, with some reports of favorable remissions [15]. Unfortunately, PEL’s rarity and poor prognosis make standardization of treatment options difficult.

## Conclusions

This case describes a woman with HIV/AIDS off ART presenting with a three-cavity pleural effusion in the setting of PEL. Her presentation supports prior evidence that increased morbidity and mortality in intracavitary PEL are positively associated with ART non-compliance, uncontrolled HIV/AIDS status, and increased body cavity involvement. Optimal therapeutic approaches are poorly understood, particularly in the context of ART non-compliance. Future research should be directed towards standardizing treatment protocols to enhance care. However, early recognition and supportive care remain essential for improving outcomes.

## References

[1] Knowles DM, Inghirami G, Ubriaco A, Dalla-Favera R (1989). Molecular genetic analysis of three AIDS-associated neoplasms of uncertain lineage demonstrates their B-cell derivation and the possible pathogenetic role of the Epstein-Barr virus. Blood.

[2] Campo E, Swerdlow SH, Harris NL, Pileri S, Stein H, Jaffe ES (2011). The 2008 WHO classification of lymphoid neoplasms and beyond: evolving concepts and practical applications. Blood.

[3] Komanduri KV, Luce JA, McGrath MS, Herndier BG, Ng VL (1996). The natural history and molecular heterogeneity of HIV-associated primary malignant lymphomatous effusions. J Acquir Immune Defic Syndr Hum Retrovirol.

[4] Schulz TF (1999). Epidemiology of Kaposi’s sarcoma-associated herpesvirus/human herpesvirus 8. Advances in Cancer Research.

[5] Brimo F, Popradi G, Michel RP, Auger M (2009). Primary effusion lymphoma involving three body cavities. Cytojournal.

[6] Hsieh PY, Huang SI, Li DK, Mao TL, Sheu JC, Chen CH (2007). Primary effusion lymphoma involving both pleural and abdominal cavities in a patient with hepatitis B virus-related liver cirrhosis. J Formos Med Assoc.

[7] Alkhasawneh A, Mohamed KS, Desai K, Omman R, Baskovich B (2022). Flow cytometric findings in primary effusion lymphoma: a report of two cases. Cureus.

[8] Grulich AE, van Leeuwen MT, Falster MO, Vajdic CM (2007). Incidence of cancers in people with HIV/AIDS compared with immunosuppressed transplant recipients: a meta-analysis. Lancet.

[9] Rabkin CS, Yellin F (1994). Cancer incidence in a population with a high prevalence of infection with human immunodeficiency virus type 1. J Natl Cancer Inst.

[10] Little RF, Gutierrez M, Jaffe ES, Pau A, Horne M, Wilson W (2001). HIV-associated non-Hodgkin lymphoma: incidence, presentation, and prognosis. JAMA.

[11] Simonelli C, Spina M, Cinelli R (2003). Clinical features and outcome of primary effusion lymphoma in HIV-infected patients: a single-institution study. J Clin Oncol.

[12] Lurain K, Polizzotto MN, Aleman K (2019). Viral, immunologic, and clinical features of primary effusion lymphoma. Blood.

[13] Moore KP, Wong F, Gines P (2003). The management of ascites in cirrhosis: report on the consensus conference of the International Ascites Club. Hepatology.

[14] Imazio M, Adler Y (2013). Management of pericardial effusion. Eur Heart J.

[15] Okada S, Goto H, Yotsumoto M (2014). Current status of treatment for primary effusion lymphoma. Intractable Rare Dis Res.

